# Elusive quality: the challenges and ethical dilemmas faced by international non-governmental organisations in sourcing quality assured medical products

**DOI:** 10.1136/bmjgh-2020-004339

**Published:** 2021-05-28

**Authors:** Katherine Enright

**Affiliations:** Nuffield Department of Population Health, University of Oxford, London, UK

**Keywords:** health policy, health systems, public health

## Abstract

Although medical products that are of sound quality are fundamental to the delivery of healthcare, so too is their availability, affordability, accessibility and acceptability. However, achieving all of these aims consistently and simultaneously may be unfeasible due to a host of barriers—no matter the country. If uncertainty, constraints and conflicting priorities also threaten their delivery, not only does the situation becomes yet more challenging, the morally just course of action becomes yet more opaque. While global health organisations, supply chains and projects are heterogenous, international non-governmental organisations (iNGOs) responding to humanitarian crises or delivering development assistance in low-income and middle-income countries are undoubtedly prone to this issue. In a novel framing of the problem of substandard and falsified medicines, this article explores some ethical dilemmas that, directly or indirectly, could result in the quality of medical products in iNGO health projects to be compromised. Drawing on a broad literature base and years of experience as a senior humanitarian pharmacist, the author reflects on the barriers, culture and system that contributes to the existence and persistence of substandard and falsified medical products in global assistance projects. The paper offers an in-depth examination of pressures that may arise in four key areas (capacity, supply chain, bureaucracy and quality assurance) and postulates on the myriad ways in which this may alter the attitudes, behaviours and decision-making of iNGOs in a manner that disincentivises the prioritisation of medical product quality. This paper does not seek to excoriate the aid sector, but rather to lend a new perspective: that such predicaments are overlooked, real-world ethical dilemmas in urgent need of greater openness, research, debate and guidance, for the benefit of moral decision-making and patient care.

**Summary box**

- Extreme but conflicting pressures in iNGO-led health programmes may mean that it is unfeasible to consistently ensure medical product availability, affordability, acceptability and accessibility, in addition to assuring quality–therefore leading to hopeless choices and ethical dilemmas.
- The context in which iNGOs operate (in terms of culture, risk, opportunity and stakeholder expectation) may disincentivise the prioritisation of medical product quality (in favour of cost or speed, for example).
- Weak capacity places a heavy toll on the sourcing of quality assured medical products due to weak regulation, underqualified staff, insufficient human resources, under-funding and scant local commodities or services.
- Supply chain complexity makes ascertaining a medical product’s provenance highly problematic and creates a landscape of ambiguous accountability.
- Excessive or ill-considered bureaucracy, in a bid to enforce accountability, may make donors (rather than beneficiaries) the most important stakeholder: thus, shifting iNGO priorities from health outcomes to grant targets.
- In-country quality assurance and control measures may be resource-heavy but inadequate; not only does this raise the issue of resource allocation, the combination of uncertainty, excessive effort and pressure to deliver, may incentivise procurement from unproven national sources.

## Introduction

Be they drugs, vaccines, therapeutic foods, medical devices, in-vitro diagnostics or assistive technologies, medical products are a core component of healthcare delivery and are fundamental to successful health interventions.^1 2^ It could be assumed then, that medical product quality is of the utmost importance in patient-centred care. However, it is not a universal certainty in health programmes that are administered by international non-governmental organisations (iNGOs)^3^ due to competing priorities, extraordinary challenges, vague accountability, a lack of accessible information and ample opportunity for sourcing products of unknown provenance. Despite the fact that development assistance for health amounted to <0.3% of global health expenditure in 2015,^4^ this is not a trivial issue. These external resources were, on average, approximately 30% of health expenditure for low-income countries, at an aggregate spend of US$19 billion.^4^

However, although iNGOs and their donors may exert significant and multifaceted influence in the countries, markets and health systems in which they operate, the nature of this is, arguably, under-researched and poorly characterised. An aspect of this, which forms the focus of this paper, is the risk of sourcing substandard and falsified medical products when quality assurance practices are compromised. Although (to the author’s knowledge) the extent to which this is a problem is unquantified; the potential risk is indisputable. iNGOs may experience numerous physical, organisational, financial, political and legal impediments when sourcing medical products for global health programmes, which creates a milieu in which uncertainty and conflicting priorities abound.

Differences in iNGO procurement practices are manifold, varying from actor to actor (eg, due to cultural or policy differences), but also within the same organisation: over time, as well as according to location, grant, type of response, capacity and personnel. Circumstances will also vary based on the medical product itself, due to attributes such as demand, available documentation, likelihood of falsification or regulatory requirements. For example, an organisation that purchases a paediatric suspension of antiretroviral medicine for a development programme in Abuja this year, will probably adopt a different approach to an emergency surgical team who were in urgent need of sterile gloves following the 2015 earthquake in Nepal. Challenges in accessing medical products are neither constant nor homogenous; however, the fact that the problem may occur at all (and, potentially, at scale) makes it worthy of analysis and general discussion.

Therefore, while it is recognised that enormous variation occurs across the humanitarian space, and examples of good procurement practice by iNGOs will be numerous, a comprehensive account of this landscape is beyond the scope of this paper. Rather, this article aims to articulate barriers that *can* occur in global health programmes. It offers an in-depth examination of pressures that may arise from such challenges and postulates on the myriad ways in which this may alter the attitudes, behaviours and decision-making of iNGOs in a manner that disincentivises the prioritisation of medical product quality. This paper does not seek to excoriate iNGOs for this, but rather to lend a new perspective: that such predicaments are actually overlooked, real-world ethical dilemmas in urgent need of greater openness, research, debate and guidance for the benefit of moral decision-making and patient care.

### A visible failure: stockouts

Pharmaceutical procurement in global health is a highly complex endeavour where achieving the ‘right’ outcome involves delivering the right goods, in the right quantity, of the right quality, to the right place, at the right time and price, and with the right information. Even in the most technologically advanced country in the world, it is exceptionally difficult to achieve all of these ‘rights’, for all products, at all times.^5^ It is therefore unsurprising that this is an unattainable goal for many health programmes in fragile contexts, as evidenced by the estimation (by MSF’s Access to Medicines campaign) that two billion people in low-income and middle-income countries (LMICs) lack access to essential medicines.^6^

The absence of medical products at the time and location that they are required can have far reaching consequences. Not only can it have a deleterious impact on the health and well-being of the patient; their family and livelihood can suffer through a loss of earnings and increased out-of-pocket expenses.^1 7 8^ If it is a routine occurrence, it may also alter health-seeking behaviour,^7^ leading patients to favour less well-regulated medical providers and drug vendors.^9^ In addition, on a macro level, it can erode public health,^8^ the reputation of the iNGO, the morale of its healthcare staff and the integrity of local pharmaceutical markets (due to demand outstripping supply, thus creating scope for substandard and falsified products).^9 10^ Therefore, there is often substantial pressure from numerous actors (including patients, caregivers, donors, governments, implementing partners and even terrorist groups, if they are health system users) to avoid or resolve stockouts with the utmost urgency.^1^

### An invisible menace: poor-quality medical products

One obvious solution for a stockout is to attempt to source the necessary medical products, or their suitable alternatives, from national vendors. This potentially avoids importation requirements, international freight costs, lengthy tenders and can be managed by those working in the field rather than relying on remote ‘head office’ procurement colleagues. Regrettably however, countries that require humanitarian assistance (for disasters *or* development) are likely to have a relatively immature regulatory capacity. It is estimated that at least 30% of National Drug Regulatory Authorities (NDRAs) globally (and the majority of countries in Africa) are not capable of conducting their core functions effectively.^11^ Unable to comprehensively quality assure medical products that are produced or imported, these countries experience a fundamental lack of safeguards for circulating healthcare goods.^12 13^ This does not, in and of itself, *cause* poor quality. However, it does provide an attractive landscape for criminal activity, commercial exploitation and lackadaisical processes in which, inevitably, substandard and falsified medical products will flourish.^9^

In extreme circumstances, substandard and falsified items represent a threat to life, result in permanent disability and contribute to antimicrobial resistance; at best they can cause self-limiting harm or are ineffective.^1 7 13^ In all cases they place a burden on healthcare resources and are a public health emergency.^7 8^ The scale of the threat is great. It is estimated that 40% of medical devices in LMICs are broken, unused or unfit for purpose^14^ and that 10.5% of drugs are substandard or falsified.^8^ (Although, as markets are not homogenous and because there is a lack of reliable evidence, it is wise to be cautious of the accuracy of these figures, which may be far higher for certain products and contexts.^15^) In high-income countries (HICs), the estimate for circulating substandard and falsified products is nearer to 1%.^15^ This disparity is undoubtedly facilitated by weak regulatory functions, and is exacerbated by paltry judicial threats, poor access to medicines and an array of ‘willing and able’ procurers.^1 8–10 12 13 16–18^

In light of these risks, it is undeniable that a commitment to sourcing quality medical products is central to the responsible and effective delivery of healthcare, as well as to the protection of public health.^1^ However, organisations operating in such contexts face the confusion that medicines and medical devices that are quality assured by the host government and are available for sale through legitimate channels may actually be poor quality. In such circumstances, the moral obligation of the iNGO may, at first, appear obvious. However, when competing priorities arise, iNGOs may struggle to reconcile responsibilities to their patients, with responsibilities to their donor(s) or host and donor State(s). Assuming that no iNGO or healthcare professional wishes to harm a beneficiary, it could be concluded then, that there are substantial forces driving any decision to renege on commitments to medical product quality. The challenges discussed below shed light on some of these forces and on the immiscible expectations of various stakeholders that can be placed on iNGO healthcare providers.

### Challenges in sourcing quality assured medical products in resource-poor settings

#### Challenges of capacity

##### The control dilemma: strengthen national systems or duplicate them?

Commonly, an iNGO will establish a parallel health supply chain for managing (planning, procuring, shipping, storing, using or disposing) its medical products.^2 19 20^ This avoids placing further strain on the national system and (perhaps more germane to an iNGO’s self-interests) it enables control of the entire value chain. This independence allows autonomy over decision-making, from provisioning to people management, which is desirable from a project management perspective. However, capacity to do so poses a sizeable challenge.

The process of crafting a demand that accurately and comprehensively reflects the needs of a population, is in budget, and can realistically be fulfilled by the vendor(s), is a formidable test of the skills and resources of an iNGO health supply chain and project team.^2^ The endeavour requires a breadth and depth of technical personnel^20^ to conduct a needs assessment, develop a formulary, analyse consumption monitoring, oversee quality assurance audits, evaluate suppliers, determine a budget or negotiate with vendors, for example. All of these broad tasks benefit from hands-on experience, formal qualifications and joined-up supply chain management software.^21 22^ Furthermore, they rely on accurate, timely and accessible communication, with clear organisational roles and responsibilities, and for the individuals themselves to form a competent and coordinated multidisciplinary team.^20^ Therefore, the human resources necessary for an effective humanitarian health supply chain can be hard to find (in sufficient number and competence)^9 19 22 23^ and thus are costly (particularly for systems that lack an end-to-end technological information management infrastructure, as is the case for many iNGOs, who may instead rely on manual monitoring, input and analysis).^21 24 25^

Additionally, the expense of purchasing, moving and managing medical products will inevitably place a heavy burden on health budgets. It is not just the items themselves that may be costly; it is the expense of shipping, storing and destroying items in accordance with Good Distribution Practices that incur substantial upfront costs, as well as requiring long-term investment.^2 25 26^ For example, warehousing temperature-sensitive medical products in a Sub-Saharan African location will likely require environmental controls and strong security measures. This kind of infrastructure may be scarce near the project site, thus requiring the iNGO to construct it, or to pay a premium for it.^9 26^ In the case of disaster-relief efforts, transport costs can be particularly high, as goods will be air-freighted for the sake of speed and the competition for space (and fuel) can be fierce.^20^

These difficulties suggest that it would be preferable for an iNGO to shift logistics responsibilities to a national system (be it public, private or charitable), at least to some degree, to achieve their project goals. Although a discussion of the options, benefits and challenges of supply chain collaboration is beyond the scope of this paper, this serves to highlight the undeniable importance of a reliable, efficient and well-functioning supply chain system (to multiple actors) for the delivery of health services.^2 19 20^ From this perspective, the common and individualistic practice of iNGOs establishing discrete supply chain systems (which require each organisation to siphon substantial funding into exclusive and impermanent (although potentially very long term) supply chain systems^20 25^) is morally questionable. Moreover, this model can encourage dependency on external resources^27^ and even undermine existing systems (eg, by poaching qualified national staff).^19^ Therefore, in this manner and somewhat perversely, iNGOs can negatively impact the countries that they seek to support.

However, unless it is the organisation’s primary purpose, *impactful* national supply chain strengthening by iNGOs is not commonplace. Perhaps this is because the use of a national system for iNGO health projects will establish the need for awkward multiagency coordination, as well as creating a confusing power dynamic.^28^ Liability, responsibility and priority may frequently be ambiguous and contested in such a relationship, in which the iNGO can *never* be an equitable partner of the host State, nor should it ever be its most valuable stakeholder (which should be its citizens). No doubt other issues such as trust, the disbursement of funds, the misalignment of political mandates and the appropriateness of iNGO vertical programmes also play a part in disincentivising collaboration on both sides.^19^ This area would benefit from further research in order to better understand barriers, scope out responsible actions for iNGOs and to progress capacity-building efforts of both national production and supply chain.

##### The competitiveness dilemma: the price of success

It is regrettable yet unavoidable that maintaining medical product quality in a low-resource setting can demand *greater* capacity.^9^ For example, health supply chains may be hampered by impassable roads, the unavailability of suitable cold chain transport and inappropriate gifting.^9 26 29^ Staff recruitment and retention can be undermined by ‘brain drain’,^19^ uncompetitive salaries and short donor contracts. The importation of goods can be frustrated by fickle, slow or ambiguous government policies, corruption and crime.^30^ The lack of capacity of Ministry of Health partners, or failing to integrate early with them, can undermine collaboration and stymie enduring change.^19^ Indeed, development assistance as a whole can be disrupted by a paucity of actionable data and insecure environments.^26^ Inevitably progress is slow, a standstill may be an achievement, and it may require a great degree of tenacity to continue to provide aid. This reality, and the modest outcomes that may be achieved in a low-resource setting, may be hard for unfamiliar donors or the public to accept, which in turn can pressurise iNGOs to reduce spending, deny supply chain costs and compromise standards in favour of demonstrable ‘quick wins’.

In aid, apart from some notable exceptions, it is an uncomfortable truth that donors (be they institutional, philanthropic or the general public) are generally unwilling to proffer the substantial financing needed for a fully funded health supply chain.^26 29^ Proposals that seek such comprehensive funding may be viewed as uncompetitive, therefore presenting charitable organisations with a moral dilemma: fail to win global health grants (thus causing financial turmoil to the organisation); or underprice the health programme (to the detriment of its delivery). The result can be a seemingly financially buoyant international charity, with restricted budgets and under-resourced health programmes. The impact of this choice is not only experienced by beneficiaries, but also places a well-documented strain on the system’s overburdened humanitarian health workers.^31 32^

Low-resource settings are, by definition, characterised by a profound lack of capacity. This places a heavy toll on the sourcing of quality assured medical products due to weak regulation, underqualified staff, insufficient human resources, underfunding and scant local commodities or services.^2 9^ The impact that this has globally, on health systems and patient outcomes, is nothing short of a public health emergency.^7 13 33^

#### Challenges of supply chain complexity

##### The inadequacy dilemma: when effort generates confidence rather than knowledge

The journey of a medical product across its life-cycle is circuitous.^8 9^ It will likely span many countries (and therefore a range of regulatory agencies and judiciaries), as well as types of vendor. Consider a medicine. The Active Pharmaceutical Ingredient and the formulation’s excipients may be manufactured in China, combined into a ‘Finished Pharmaceutical Product’ in India, labelled in Senegal, packaged in Malaysia, purchased in France via a Canadian broker, distributed under the control of a Netherlands procurement agency, and imported to Vietnam. From here the product may be administered at an iNGO’s project site; or it may travel through the hands of numerous additional national vendors, pharmacies, agents, distributors and hawkers before reaching the patient.

If a procurer seeks to purchase this medicine in-country, the chief problem (in quality terms) will likely be an opaque supply chain.^34^ Ascertaining a product’s provenance, as well as how it has been stored and handled since manufacture, can be unfeasible for an iNGO due to a lack of reliable or validated information.^9^ Where this is the case it is highly problematic, because if this critical information is unavailable or questionable, then a medical product cannot be quality assured—no matter how many resources are brought to bear to evaluate it. This concept can be challenging for stakeholders to accept, particularly if resources (which may be substantial in terms of time, money and personnel) are expended to evaluate the national market or downstream supply chain. It would be interesting to explore whether Festinger’s Cognitive Dissonance theory^35^ applies in these circumstances: to research whether the effort of burdensome ‘local market assessments’ causes an increase in the subjective value of national vendors by iNGOs.

An iNGO may complement quality assurance activities with product testing (in a laboratory or the field) to gain confidence in the quality of goods and, by extension, the integrity of the supply chain. This *may* flag suspected poor-quality medical products; however, it is unlikely that the nature or cause will be unequivocally detected due to the unfeasibility of utilising powerful laboratory technologies, and the inherent limitations of field testing.^10 13 15 33^ It is also improbable that the procurer will have the means to both identify the responsible global actor *and* to hold them to account, given that pharmaceutical regulation and judicial enforcement would be the responsibility of differing governments.^9 13 36^ Furthermore, null results do not necessarily indicate quality when conducted in isolation.^13^ Despite these points, and the fact that product testing is potentially complex, expensive, destructive^8 15^ and, for some items, inadequate (particularly medical devices) it is an option for (and employed by some) iNGOs. How quality control can impact attitudes and decisions in these organisations is worthy of research, to help inform policy positions on the role of testing in organisational quality and risk management.

These facets of supply chain complexity create a landscape in which accountability for product quality is highly uncertain.^2 9^ This may encourage national purchasing, which is particularly problematic for procurers whose resources and buying power are inadequate for protecting them from unscrupulous activities in the marketplace. Regrettably, the potential for such activity on the global pharmaceutical market is enormous. As well as being critical to every individual on the planet for reaching and sustaining the best possible quality of health, it is one of the world’s largest by sales (the IQIVA Institute for Human Data Science anticipate that it will exceed US$1.5 trillion by 2023).^37^ With a broad and consistent base of consumers, as well as the opportunity for (frequently considerable) income generation, it is unsurprising that this global market is complex, diverse and attracts exploitation: from petty corruption through to serious organised crime.

#### Challenges of bureaucracy

##### The accountability dilemma: should the piper serve the pauper or the payer?

Fiscal responsibility and accountability are exceptionally important in the aid sector, where tracking and justifying spend to donors and governments is a legal requirement and a moral obligation.^38^ However, the policies, procedures and *culture* that exist in order to achieve this level of scrutiny are not always the most advantageous for beneficiaries.^26 29^

First, funds are often earmarked to specific activities and spend categories by donors (the impact of which has received attention in the scientific community).^29 39 40^ This can create confusion, inefficiencies and undermine an iNGOs ability to autonomously adapt to the unpredictable context in which they work.^29^ Second, funds may be released on the day that a project goes live,^26^ even though activities such as recruitment and pharmaceutical procurement can have a very long lead time. It could take many months to prepare, adjust, ship and import a consignment of medical products to a programme in Myanmar or Afghanistan, for example. Therefore, for new programmes, the iNGO may face the dilemma of whether to scale back targets based on the new timeframe (which may bring about financial penalty from the donor), or to purchase medical products from a more accessible location (though one that is unlikely to comply with donor quality assurance policy).

Third, harsh penalties for overspend or underspend (such as disallowances and reputational damage) can have a significant and long-lasting detrimental impact to the project, staff and organisation. This encourages an iNGO to prioritise adherence to a budget that, because it is fixed (or difficult to modify), offers little reward for optimising value for money and the responsible use of resources. As drugs and medical devices often consume a large percentage of a health budget, it is an attractive area to make savings or to absorb increased spending. This can have a particularly pronounced effect on medical product quality. For example, in the case of overspend, slashing the drugs budget without altering project targets will often make international procurement unfeasible. This may force sourcing from the national market where, in humanitarian or development settings, the risk of substandard and falsified items is high.^8 41 42^ Underspend on the other hand may motivate an iNGO to purchase surplus medical supplies at the close of the project to balance accounts and secure a favourable donor assessment. As speed of delivery and accessibility are the primary concern in this instance, national vendors would be preferable to a time-consuming international order (which may also attract the support and attention of head office procurement teams).

Fourth, stringent policies and key performance indicators may be designated by donors to safeguard and monitor project quality, but instead can detract from it.^10^ Policies demanding that iNGOs consistently meet the standards of HICs may be attractive, as they are comparatively straightforward to write and understand, are familiar (being akin to the donor’s national legislature) and, reassuringly, do not permit quality to be compromised. However, if unachievable, actors may find that loopholes and waivers are easier to realise than quality assurance risk mitigation measures. Likewise, indicators that use easy-to-interpret quantitative measures (eg, stock levels) may unintentionally encourage product quality to be compromised, if not balanced with reasonable proxies for it. The latter, however, are more difficult to obtain, harder to understand and are not as directly relatable.

Therefore, for humanitarian and development contexts as a collective, an inflexible approach is not realistic —nor is it evidence-based. Although simple, standardised procedures and indicators may be suitable for some commodities, the complexity of sourcing medical products for use in LMIC contexts demands a more detailed, resourced and discerning method of oversight. Systems that fail to legislate for circumstances in which international procurement is unfeasible (eg, emergencies and supply chain failures), or who make excessive concessions through the use of waivers, will leave the iNGO without guidance for navigating procurement dilemmas and will encourage an all or nothing approach to medical product quality. Furthermore, in practice it may reinforce an unhelpful ‘us and them’ mentality in which ‘local procurement’ is synonymous with high-risk, poor-quality medical products. This can diminish respect for national regulatory systems, create markets that are excessively ‘Western’ focused (as exemplified by price hikes for EU Good Manufacturing Practice compliance^10^ or the imitation of CE marks^43^), and create protocols that are in contention with national importation requirements.

Strict protocols, basic indicators, written reports and the threat of sanctions are commonly used by institutional donors as tools for evaluating performance and creating accountability. This approach may be because, like iNGOs, donors may lack the resources required to create sufficiently nuanced policies and indicators, or the sophistication to effectively implement them. While a simplistic cookie-cutter approach may be advantageous for donor resources, it can undermine collaboration with iNGOs and disincentivise a commitment to quality.^44^ When this power imbalance is entrenched by harsh reprisals for deviation from procedures or expected targets, it can further discourage candid dialogue and disempower iNGO adaptability.^44^ As a result, there is a risk of establishing a tick-box culture that focuses on donor compliance, rather than health outcomes. Further research to appraise the impact of donor rules on organisational decision-making, medical product quality and, ultimately, health outcomes is strongly recommended.

#### Challenges of quality assurance

##### The uncertainty dilemma: when quality is a lottery

Unfortunately, the quality of a medical product cannot be determined by its appearance.^15^ Nor can it be reliably and routinely guaranteed, at scale, solely via chemical analysis.^9 13^ Though not without limitations, a procurer’s confidence in product quality is either based on trust in the NDRA,^45^ or based on knowledge and evaluation of the manufacturer’s, distributor’s and supply chain’s system and quality assurance measures.^46^ If these are robust and complete then, logically, the quality of the product can be consistently guaranteed.^47^ However, substantial money, skill and experience (both technical and people related) are required in order to visit and inspect infrastructure, equipment, materials, staff, procedures, permissions and records of the company.^10 12 46^ This is evidenced by the fact that only the results of specific regulators (either individuals or organisations) will be acknowledged as credible by global regulatory institutions (eg, the Pharmaceutical Inspection and Co-operation Scheme) and most NDRAs.

The issue of quality variance is clouded by the fact that its classification is somewhat subjective.^41 48^ This is not necessarily due to a deviation of standards between countries, organisations or individuals (although this may be the case), it can be influenced by circumstances during evaluation.^7^ Differences in capacity, competence and ability to access key locations may alter how thoroughly an assessor (eg, a regulatory authority or iNGO health provider) is able to scrutinise a manufacturer, distributor or product.^12 49^ In addition, the experience and risk appetite of the assessor (or their organisation) may impact their evaluation of what is deemed to be acceptable. As a result, an item that is quality assured by one country, donor, charity or healthcare professional may not be approved by another, even though all parties are referencing the same standards.

If the quality assurance of medical products is an institutional responsibility that yields differing conclusions, this begs the question: who *should* determine what is acceptable? This moral dilemma is faced by iNGOs involved with health provision in LMICs, if a medical product that is legally approved by the host nation would be rejected by the benefactor’s one. In 2010 a WHO assessment of regulatory systems in Sub-Saharan Africa concluded that ‘on the whole, [the assessed] countries did not have the capacity to control the quality, safety and efficacy of the medicines circulating on their markets or passing through their territories’.^50^ Today the global differential is still alarming: it is estimated that nearly three quarters of NDRAs are benchmarked at Maturity Level 1 or 2, and are therefore not considered to have a ‘stable, well-functioning and integrated regulatory system’.^51^ Currently there is a lack of consensus (and a paucity of dialogue) regarding whose medical products, in a generalised sense, should be sourced: those legally approved by the host nation (but whose quality may be questionable), or those equivalent to the standards of the benefactor’s nation (where, in this paper, it is assumed that the latter is a WHO-listed authority benchmarked at Maturity Level 3 or 4).

##### The unspeakable dilemma: choosing quality or availability

For a health iNGO, there are several options regarding a quality assurance audit of suppliers in LMIC settings, including perform them in-house, collaborate with other organisations, rely on publicly available reports, outsource them to a third-party procurement agent or do not conduct them. (These choices are discussed briefly below.) However, it is feasible that none of these options will be ideal, even in combination, particularly for programmes with a broad medical product list.

In-house quality assurance is prohibitively expensive for many iNGOs, given the plethora of medical products, suppliers and destinations involved. However, larger agencies may consider this worth the effort for key countries, programmes or where collaborative opportunities exist. Invariably however, the audits do not deliver a holistic package of knowledge on which to base all sourcing decisions for the project. This is because they may only give an ad hoc snapshot, be insufficiently in-depth, fail to cover all required products or provide technical information that the organisation is unable to interpret or action.

Although a staple method for NDRAs (and one that is encouraged by the WHO),^45^ a ‘reliance’ approach to quality assurance is unfeasible for many iNGOs. This technique involves establishing equivalence of each batch of a medical product with one that is approved by a ‘trusted’ NDRA, or auditor. This desk-based assessment may be advantageous in terms of cost and personnel security but, as it involves a review of dossiers and documentation for each individual product, it is dependent on access to commercially sensitive information (and staff with the skill to interpret it). Ideally, this is achieved via cross-organisational information-sharing agreements, but iNGOs may lack the negotiating power (that NDRAs enjoy) to broker such an exchange. Instead therefore, they may need to piece together data from public domains (eg, the list of European Medicines Agency-approved manufacturers), the host government, partner organisations and the manufacturer themselves. Not only is this time-consuming, it is unlikely to yield the sufficiently detailed or validated information that is necessary for evidence-based decision-making.

Furthermore, iNGOs that require a range of commodities may prefer wholesalers who are a ‘one-stop-shop’ and may, therefore, deem the evaluation of individual production lines of a manufacturer to be unworkable (or pointless, if the minimum order quantities are greater than their needs). Thus, based on the same concept of trust and reliance, charities with the risk appetite to do so may conduct remote assessments of vendors (not individual products) using shared or publicly obtainable information. As the procurement habits of a humanitarian organisation are unlikely to overlap with that of an HIC, data from a stringent and reliable NDRA (ie, benchmarked at Level 1 or 2) are in short supply. Reports of a trusted auditor (eg, QUAMED) may be available but are unlikely to yield positive results,^42^ or it may only be possible to gather several pieces of vague and decontextualised data—a list of the vendor’s customers, for example. This practice, and the uncertainty it creates, may increase the risk of sourcing poor-quality medical products and may even invite exploitation.^9 10^ Given the risks, this area would benefit from scrutiny to understand the extent of (and drivers for) remote assessments and whether poor-quality data have an influence on iNGO decision-making.

For many organisations, purchasing will either be outsourced to an international procurement agent or conducted nationally (either by internal personnel, or integrated with a partner’s supply chain). In the case of the latter (national procurement), the iNGO may purely consider their role to be programme implementation and argue that product quality is a government responsibility. Whereas in the former circumstance, a supplier is relied on to ensure quality standards are met. Anecdotal evidence suggests that organisational culture, risk appetite, capacity, multinational status and operational constraints are all influential in determining whether an NGO considers product quality to fall under its responsibility. Further research would be beneficial to qualify this, comprehend the role of donors and to unravel key aspects of organisational decision-making.

Given the significant level of effort required to conduct quality assurance activities, an ethical issue that is common in healthcare comes to the fore: that of resource allocation. In the event of supply chain delays or failures, iNGO actors in humanitarian and development settings may be unable to source goods in both the quality and quantity required (with the time and funds available). This may cause considerable internal conflict regarding budgets and resource allocation. This tension is, frequently, a matter of quality versus availability.^52^ Broadly, for an iNGO to guarantee medical product quality in a low-income country, expensive and time-consuming international procurement (which depletes significant funds, time and effort from a limited pool of resources) is required. Not only does this divert capital and people from other needs and initiatives, it may be impracticable for emergencies and short-lived grants or contracts. On the other hand, availability can often be comfortably achieved through the indiscriminate purchase of goods from a market according to cost, ease and speed. While this may be a highly attractive course of action, it bypasses quality assurance and therefore threatens medical product safety and efficacy (and, by extension, patient well-being).

This is an ethical challenge that, as yet, has been largely unexplored. This may be because, culturally, only one course of action in this dilemma is considered acceptable. The idea of compromising a medical product’s quality, safety and efficacy, and distributing it to a vulnerable (perhaps paediatric) beneficiary, seems unconscionable. (Indeed, arguments that this runs contrary to the tenets of beneficence, non-maleficence and justice have been presented^53^). Therefore, discussion of this issue tends to focus on the need for quality: how it should be nurtured, methods for monitoring it and ways to enforce it. While this line of enquiry is important, it alienates those facing current and real-world challenges. It fails to acknowledge the competing pressures that urgency and budgets create, instead establishing a paradigm where deviation from a stringent quality standard becomes an unreasonable and unspeakable action.

This, of course, does not solve the issue—rather, it compounds it. If an iNGO chooses to derogate a procedure that could impact medical product quality, it forces the decision to be made internally (and the donor or State may not even be involved). Away from the public domain, the global health community is unable to compare, scrutinise and debate purchases (or the consequences of them), and thus cannot learn from them. Not only is this conducive to repeated errors of judgement (and therefore potential beneficiary harm), it impedes collective work towards an evidence-based framework for handling these tough decisions. Therefore decision-makers and influencers of iNGO health programmes urgently need to concede that this is a problem, and to create an environment that facilitates open and honest dialogue.

## Conclusion

The complexity of medical products (as well as the systems in which they exist) and the limits of technology, combined with a dearth of capacity in a context that demands increased resources (due to natural and man-made disruptions), creates a colossal barrier to sourcing quality assured medical products. For iNGO procurers, these challenges are compounded by the numerous additional (and oftentimes) competing donor, host and user expectations. When these pressures are nested in a culture of weak accountability and unsympathetic reprisals, the reward for compromising medical product quality and achieving competing priorities may outweigh the risks, costs and challenges associated with adhering to legal and moral quality assurance requirements.

Despite the challenges discussed in this paper, it is vital that the global health community is undeterred in its mission to eradicate the scourge of falsified and substandard medical products that threaten patient health. However, *because* of these challenges, it is also vital that the global health community openly explores justifications and risk-managed methods for compromising medical product quality. Greater research is required to gather and analyse the normative values of actors involved in the provision and consumption of medical products, and to understand and categorise the influences and influencers at play. This initial step should facilitate conversation regarding the circumstances that would willingly lead to, through action or inaction, the failure to assure that medical products distributed within the supply chain meet minimum quality standards. Accepting and understanding these decisions is a prerequisite to designing solutions that will help to avoid them in the future but, more than this, is also key to establishing the sector-wide professional empathy that is necessary for acknowledging difficulties, encouraging honest dialogue and improving standards.

## Data Availability

There are no data in this work
